# Radical Chemistry in a Femtosecond Laser Plasma: Photochemical Reduction of Ag^+^ in Liquid Ammonia Solution

**DOI:** 10.3390/molecules23030532

**Published:** 2018-02-27

**Authors:** Victoria Kathryn Meader, Mallory G. John, Laysa M. Frias Batista, Syeda Ahsan, Katharine Moore Tibbetts

**Affiliations:** Department of Chemistry, Virginia Commonwealth University, Richmond, VA 23220, USA; meadervk@vcu.edu (V.K.M.); johnmg@vcu.edu (M.G.J.); friasbatistlm@vcu.edu (L.M.F.B.); ahsansn@vcu.edu (S.A.)

**Keywords:** femtosecond laser, radical scavenger, hydrogen peroxide, liquid ammonia, peroxynitrite, metal nanoparticle

## Abstract

Plasmas with dense concentrations of reactive species such as hydrated electrons and hydroxyl radicals are generated from focusing intense femtosecond laser pulses into aqueous media. These radical species can reduce metal ions such as Au^3+^ to form metal nanoparticles (NPs). However, the formation of H_2_O_2_ by the recombination of hydroxyl radicals inhibits the reduction of Ag^+^ through back-oxidation. This work has explored the control of hydroxyl radical chemistry in a femtosecond laser-generated plasma through the addition of liquid ammonia. The irradiation of liquid ammonia solutions resulted in a reaction between NH_3_ and OH·, forming peroxynitrite and ONOO^−^, and significantly reducing the amount of H_2_O_2_ generated. Varying the liquid ammonia concentration controlled the Ag^+^ reduction rate, forming 12.7 ± 4.9 nm silver nanoparticles at the optimal ammonia concentration. The photochemical mechanisms underlying peroxynitrite formation and Ag^+^ reduction are discussed.

## 1. Introduction

The chemistry of short-lived radical species such as $e_{aq}^{-}$ and OH· in aqueous solutions has a wide applicative scope. This radical chemistry is integral to the understanding of radiation-induced damage in biological systems [1,2], the development of radiation-based strategies for the removal of environmental pollutants [3,4,5], and the design of environmentally friendly synthesis routes to metal nanoparticles (NPs) [6,7,8,9,10]. Most aqueous radical chemistry studies use ionizing radiation to induce radical formation [1,2,3,6,7,8,9], but the same radical species can be generated by focusing ultrashort laser pulses into aqueous media. Sufficiently intense pulses induce multiphoton ionization of water molecules, forming a dense, localized plasma, in a process called optical breakdown (OB) [11,12]. The formation of OB plasma in water includes several reactions, not limited to the following [13,14,15,16,17,18]:

(1) $$H_{2}O\overset{nh\nu }{\to }e^{-}+H^{+}+\mathrm{OH}\cdot$$

(2) $$e^{-}\to e_{\mathrm{aq}}^{-}$$

(3) $$H_{2}O\overset{nh\nu }{\to }H\cdot +\mathrm{OH}\cdot$$

(4) $$2\mathrm{OH}\cdot \to H_{2}O_{2}$$

(5) $$H\cdot +H_{2}O\to {H_{3}O}^{+}+e_{\mathrm{aq}}^{-}$$

These reactions enable the photochemical reduction of metal ions in solution, making metal NPs without chemical reducing agents [18,19,20,21,22,23,24,25,26,27,28,29]. In particular, high concentrations of both immediately formed free electrons (10^20^ to 10^22^ cm^−1^) [12] and subsequently formed hydrated electrons (up to 0.1 M) [17] in OB plasmas enable efficient metal ion reduction, even in air-saturated solutions, where O_2_ acts as a scavenger [30]. Both hydrated electrons and H_2_O_2_ are well suited to the photochemical conversion of Au^3+^ ions to gold nanoparticles (AuNPs), because of their predisposition towards autocatalytic reduction [27,28,29]. However, H_2_O_2_ formation hinders the application of this technique to other metals, because H_2_O_2_ is a strong oxidant. For instance, Ag^0^ back-oxidizes to Ag^+^ in the presence of H_2_O_2_ [6,31,32], inhibiting silver nanoparticle (AgNP) formation by Ag^+^ photochemical reduction in an OB plasma [20,24].

The back-oxidation of Ag^0^ to Ag^+^ in the presence of H_2_O_2_ is well known; many γ radiation methods prepare AgNPs from Ag^+^ ions by using secondary alcohols as hydroxyl radical scavengers [9] or stabilizers [6,9]. Ethylenediaminetetraacetic acid (EDTA) has also been reported to help Ag^+^ ion reduction under γ irradiation, where Ag^+^ ions complex to the carboxylate groups in EDTA [7,33]. The preparation of AgNPs in OB plasma is made possible by adding other species to the Ag^+^ precursor solution, including TiO_2_ [34], sodium citrate [35], and sodium dodecylsulfate (SDS) surfactant [36]. The laser ablation of silver targets immersed in electrolyte solutions has also yielded colloidal AgNPs [37], although the AgNPs formed by laser ablation can also be back-oxidized in OB plasma [31]. An additional challenge to forming AgNPs from AgNO_3_ with radiation-based methods may lie in the activity of the nitrate ion as a hydrated-electron scavenger [30,38]. For this reason, a number of studies used AgClO_4_ as their Ag^+^ AgNP precursor in radiolysis experiments [39,40,41]. Recently, colloidal AgNPs were synthesized from aqueous AgNO_3_ solutions containing polyvinylpyrrolidone (PVP) in OB plasma, but only when ammonia was present in the solutions [24]. Earlier studies have reported complexation of the Ag^+^ ion with ammonia to form Ag(NH_3_)_2_^+^ [40,42], which the authors of [24] attributed as the ability to form AgNPs. The lowered reduction potential of Ag(NH_3_)_2_^+^ compared to free Ag^+^ [40] suggests that manipulation of the NH_3_ concentration may enable control over both the formation rate and the resulting properties of AgNPs.

Silver’s microbial properties have motivated studies into silver nanostructure preparation and the incorporation of silver nanostructures into medical devices [43,44,45]. The unique optical properties of colloidal silver also make it a cost-effective surface-enhanced Raman spectroscopy (SERS) substrate option as compared to AuNPs [35,46]. In this article, we aim to understand the radical chemistry leading to AgNP formation when aqueous solutions of ammonia and AgNO_3_ are irradiated by strong-field, ultrashort laser pulses. We provide evidence of the major radical-mediated reactions and products formed in OB plasma, including peroxynitrite and ONOO^−^, from the irradiation of different concentrations of aqueous ammonia solution in an OB plasma. Neither AgNO_3_ nor AgClO_4_ solutions produced AgNPs in the absence of ammonia. In the presence of ammonia, the irradiated solutions of both AgNO_3_ and AgClO_4_ showed that the growth kinetics and final sizes and morphologies of the AgNPs depended on the ammonia concentration. Understanding the radical-mediated reactions involved will further the development of other laser and γ radiation-based synthesis techniques for metal NPs.

## 2. Experimental Methods

### 2.1. Materials

Silver nitrate, AgNO_3_ (Acros, Fair Lawn, NJ, USA), silver perchlorate, AgClO_4_ (Fisher Scientific, Fair Lawn, NJ, USA), sodium nitrite, NaNO_2_ (Fisher), potassium nitrate, KNO_3_ (Fisher), potassium hydroxide, KOH (Fisher), hydrogen peroxide, H_2_O_2_ (30%; Fisher), and ammonia solution, NH_3_ (32%; Emplura, Billerica, MA, USA) were used without further purification and were made into stock solutions using Milli-Q filtered water (18 MΩ cm^−1^). Titanium dioxide powder, TiO_2_ (Sigma Aldrich, St. Louis, MO, USA) and sulfuric acid, H_2_SO_4_ (Fisher) were used without further purification.

### 2.2. Sample Preparation

AgNPs were prepared by irradiating precursor solutions containing 0.1 mM Ag^+^ in the form of either AgNO_3_ or AgClO_4_, in the presence of ammonia (0–20 mM). The Ag^+^ and ammonia working solutions were prepared from stock solutions directly in the cuvettes immediately before irradiation.

Experiments testing the amount of H_2_O_2_ produced in the OB plasma were carried out by irradiating water or water with different concentrations of ammonia (0–600 mM). The ammonia was added from a stock solution directly into the cuvette; this was immediately followed by irradiation (60–600 s). Once the irradiation was complete, 400 µL of titanium(IV) sulfate (25 mM) was added to the cuvette, and an absorption spectrum was recorded. More details on the quantification of H_2_O_2_ by titanium(IV) sulfate are provided in Section 2.4.

The synthesis of peroxynitrite was adapted from [47], in which NaNO_2_ (0.58 M) was added to a stirring solution of H_2_O_2_ (0.31 M) and H_2_SO_4_ (0.15 M), followed by the immediate addition of KOH (2.5 M).

### 2.3. Instrumentation

The experimental setup has been described previously [29]. Briefly, a titanium–sapphire chirped-pulse amplifier (Astrella, Coherent, Inc., Santa Clara, CA, USA), delivering 5 mJ, 30 fs pulses, with the bandwidth centered at 800 nm and a repetition rate of 1 kHz, was used. The pulse energy was adjusted with a zero-order λ/2 waveplate (ThorLabs, Inc., Newton, NJ, USA) and a broadband thin-film polarizer (Altechna, Vilnius, Lithuania) to 1 mJ. The laser beam was expanded from 11 to 29 mm prior to focusing with an f=5 cm aspheric lens, to produce a focal beam waist of 6.5 µm and a peak intensity of 2.5 × 10^16^ W cm^−2^. The solutions were irradiated for times ranging from 60 to 600 s, depending on the experiment. To monitor the reaction kinetics during irradiation, the experiments were performed in a home-built in situ UV-visible spectrometer, consisting of a stabilized deuterium–tungsten light source (Ocean Optics, DH2000-BAL, Winter Park, FL, USA), optical fibers, two pairs of off-axis parabolic mirrors, and a compact spectrometer (Ocean Optics, HR4000).

### 2.4. Characterization

#### 2.4.1. Quantification of H_2_O_2_

Hydrogen peroxide concentrations were quantified following the method in [48]. A solution of titanium(IV) sulfate (25 mM) was prepared by digesting a weighed amount of TiO_2_ in concentrated H_2_SO_4_ for 16 h at 170° and diluting with water once cooled to room temperature. Titanium(IV) sulfate (Ti^4+^) reacts with H_2_O_2_ according to

(6) $${\mathrm{Ti}}^{4+}+H_{2}O_{2}+2H_{2}O\to \mathrm{Ti}O_{2}H_{2}O_{2}+4H^{+}$$

to form pertitanic acid, TiO_2_H_2_O_2_, which absorbs at 407 nm with the intensity directly related to its concentration [29,48]. The H_2_O_2_ formed in the irradiated solutions was quantified against a calibration curve, which was constructed by adding titanium(IV) sulfate to different concentrations of standardized H_2_O_2_ solutions; all solutions contained 3.3 mM titanium(IV) sulfate. The H_2_O_2_ solutions were standardized by titrating with KMnO_4_ [49], which itself was standardized by titrating weighed amounts of sodium oxalate, following the procedure from [50]. Further details of this calibration step can be found in [29].

#### 2.4.2. Transmission Electron Microscopy (TEM)

AgNP images were collected using TEM (JEOL JEM-1230). Colloidal AgNPs were drop-casted onto a carbon-coated grid (Ted Pella, Inc., Redding, CA, USA) and left to dry for 24 h or longer. ImageJ software was used to determine particle sizes.

#### 2.4.3. Ion Chromatography (IC)

The chromatographic equipment consisted of a DIONEX ICS-1000 ion chromatogram coupled to a mass spectrometer (ThermoFisher Scientific, Fair Lawn, NJ, USA). Chromatographic separations were carried out using an AS14A column (maintained at 30 °C) and an AS14 guard column running through an AERS 500 4 mm suppressor. The eluent was 8 mM aqueous sodium bicarbonate and 1 mM aqueous sodium carbonate, the flow rate was 1.2 L/min, and the injection volume was 0.5 µL. All measurements were performed at room temperature.

## 3. Results

### 3.1. Irradiation of Aqueous Ammonia Solutions

**Figure 1:**
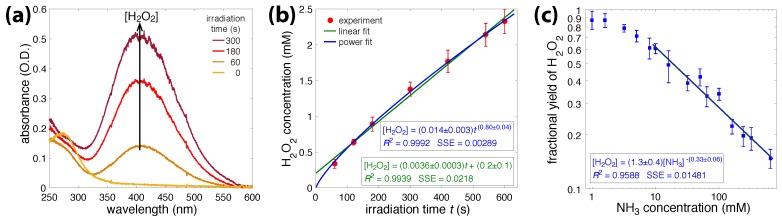
(**a**) Absorption spectrum of TiO_2_H_2_O_2_ complex formed after adding titanium(IV) sulfate to irradiated water; (**b**) H_2_O_2_ concentration vs irradiation time. Experimental data points (red) fit with with linear (green) and power (blue) functions; (**c**) Log–log plot of fractional H_2_O_2_ yield vs. NH_3_ concentration with linear fit a 10–600 mM NH_3_ range. Error bars in (**b**,**c**) denote standard deviation over four independent experiments.

To obtain the baseline rate of H_2_O_2_ formation under our experimental conditions, water samples were irradiated for times ranging from 60 to 600 s, and titanium(IV) sulfate was added to the samples afterwards. Figure 1a shows the absorption spectrum of TiO_2_H_2_O_2_ formed under the specified times (0–300 s). The increased absorbance at 400 nm with irradiation time reflected the production of H_2_O_2_ [29,48], which was produced in higher quantities as the irradiation continued. Figure 1b shows the H_2_O_2_ concentration as a function of the irradiation time. The experimental data were fit to both linear (green) and power (blue) functions, by nonlinear least-squares methods. The power law gave a closer fit, demonstrating that the H_2_O_2_ concentration grew relative to time as $t^{0.8}$. The sublinear growth in the H_2_O_2_ concentration may have resulted from H_2_O_2_ molecule fragmentation in the laser plasma or the reaction of hydrogen peroxide with additional OH· radicals, or possibly both.

To quantify radical-scavenging properties of ammonia, NH_3_ solutions (1–600 mM) were irradiated for 300 s, and the H_2_O_2_ concentrations were quantified with titanium(IV) sulfate. The fractional yields of H_2_O_2_ produced at different NH_3_ concentrations, relative to the irradiation of pure water for 300 s, are shown in Figure 1c, and the associated numerical values are reported in Table S1 of the supporting information. A linear fit to the log–log data, in the range of 10 to 600 mM NH_3_, resulted in the relationship $\left[H_{2}O_{2}\right]∼{\left[{\mathrm{NH}}_{3}\right]}^{-0.33}$. This result shows that ammonia reacts with OH· radicals or H_2_O_2_, or both, and that the quantity of H_2_O_2_ produced shrinks as the ammonia concentrations increase.

**Figure 2:**
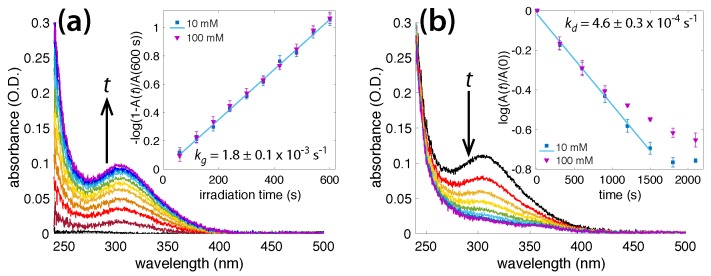
(**a**) Absorbance spectra taken every 60 s during irradiation of 10 mM NH_3_ solution. Inset: Equation (7) plotted for 10 and 100 mM NH_3_ solutions vs irradiation time, with linear least-squares fit; (**b**) Absorbance spectra taken every 300 s following termination of initial 600 s laser irradiation of 10 mM NH_3_ solution. Inset: Equation (8) plotted for 10 and 100 mM NH_3_ with linear least-squares fit.

In solutions containing at least 10 mM NH_3_, a peak centered around 302 nm appeared and increased in intensity with irradiation time, and subsequently disappeared once irradiation was terminated. Figure 2a shows the absorbance spectra collected every 60 s during the irradiation of a 10 mM NH_3_ solution. A growth rate constant, $k_{g}$, could be calculated using the slope

(7) $$-\log_{10}\left(1-\frac{A(t)}{A(600\;s)}\right)=k_{g}t,$$

where A(*t*) is the 302 nm absorbance at time *t* (in seconds), and A(600) is the 302 nm absorbance after 600 s. To prevent the quantity on the left side of Equation (7) from approaching infinity as t→600 s, the reference quantity A(600) was taken to be the mean value plus the standard deviation obtained over four experiments. The inset in Figure 2a shows a plot of Equation (7) over time, for 10 and 100 mM NH_3_ solutions, with a growth rate constant value of $k_{g}$ = 1.8 ± 0.1 × 10^−3^ s^−1^. Raising the NH_3_ concentration above 10 mM did not affect the growth rate; the absorbance values in the Figure 2a inset overlap for both the 10 and 100 mM ammonia solutions. This suggests that the growth of the 302 nm peak was zeroth order with respect to NH_3_, within the concentration range of 10–100 mM.

When the laser irradiation stopped, the 302 nm absorbance peak disappeared slowly. Figure 2b shows the spectra of the 10 mM ammonia solution, collected every 300 s after the initial 600 s irradiation. The decay rate constant, $k_{d}$, was calculated by extracting the slope of

(8) $$\log_{10}\left(\frac{A(t)}{A(0)}\right)=k_{d}t,$$

where A(*t*) A(0) is the 302 nm absorbance immediately following irradiation. The inset in Figure 2b shows the decaying 302 nm peak for 10 and 100 mM NH_3_ solutions. The linear region (1000 s for 100 mM and 1500 s for 10 mM NH_3_) shows a decay rate constant value of $k_{d}$ = 4.6 ± 0.3 × 10^−4^ s^−1^. The most likely chemical species behind this 302 nm peak was peroxynitrite, ONOO^−^, which is known to absorb at 302 nm [51,52]. A further discussion of the radical reactions leading to the formation of this transient product is presented in Section 4.

### 3.2. Photochemical AgNO_3_ Reduction in Liquid Ammonia Solutions

In the absence of NH_3_, AgNP formation in the femtosecond laser-irradiated AgNO_3_ solutions was unreliable, and generally no AgNPs were formed at all (Supporting Information, Figure S1a), as was consistent with previous results [24]. Any particles that did form quickly agglomerated and precipitated out of the solution. Hydrogen peroxide oxidized Ag^0^ back to Ag^+^ [6,31,32], disrupting photochemical AgNP synthesis [20,24]. This process was evident from the decreased amount of H_2_O_2_ produced by the irradiation of a 0.1 mM AgNO_3_ solution, as compared to pure water (Supporting Information, Figure S1b). Adding NH_3_ to the AgNO_3_ solution enabled AgNP formation, for which both the growth rate and AgNP properties were highly dependent on the NH_3_ concentration.

**Figure 3:**
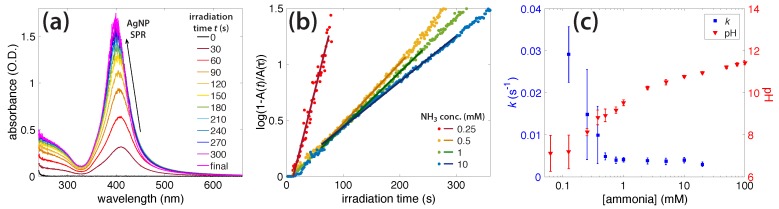
(**a**) Absorbance peak of silver nanoparticles (AgNPs) in 1 mM NH_3_ as it grew during irradiation; (**b**) AgNP formation rates in different concentrations of ammonia; (**c**) Rate constant as a function of NH_3_ concentration, overlaid with the initial pH of Ag–NH_3_ solutions.

Figure 3a shows the growth of the AgNP surface plasmon resonance (SPR) peak at around 400 nm with the irradiation time for a solution containing 1 mM NH_3_. The cessation of the peak growth at 300 s of irradiation indicated the complete conversion of Ag^+^ to AgNPs. Figure 3b shows the rate of AgNP SPR growth in different NH_3_ concentrations as a semi-log plot, according to Equation (7). The growth rate constant *k* was extracted from the slope of the least-squares fit line, according to Equation (7). The fastest growth occurred with the lower concentrations of NH_3_, and, as more was added, the formation kinetics slowed. Figure 3c shows both the AgNP growth rate *k* and initial solution pH as functions of the NH_3_ concentration; numerical values are given in the supporting information in Table S2.

### 3.3. Characterization of AgNPs

**Figure 4:**
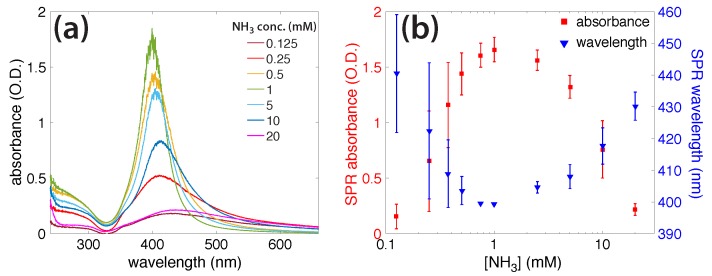
(**a**) Absorbance spectra of silver nanoparticles prepared with different amounts of NH_3_, labeled; (**b**) Surface plasmon resonance (SPR) absorbance (red, left) and SPR wavelength (blue, right) as function of NH_3_ concentration.

Figure 4a shows the final AgNP absorbance spectra, synthesized in different concentrations of NH_3_. At 0.25 and 10 mM, the formation kinetics were respectively fast and slow with respect to the kinetics of the 1 mM solution (Figure 3b). The SPR absorbance at both 0.25 and 10 mM NH_3_ concentrations were less intense, red-shifted, and broader, as compared to the SPR absorbance at 1 mM, suggesting that larger NPs were formed and suggesting possible particle agglomeration. Figure 4b shows the relationship between the AgNP SPR absorbance and wavelength for each of the NH_3_ concentrations. The SPR peak is most intense and blue-shifted at 1 mM NH_3_, implying that this concentration was optimal for producing small, monodisperse AgNPs.

**Figure 5:**
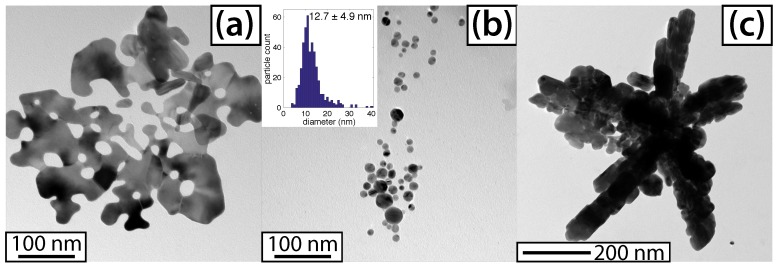
Transmission electron microscopy (TEM) images of silver nanoparticles, with (**a**) 0.25; (**b**) 1; and (**c**) 10 mM NH_3_. Histogram overlay corresponds to (**b**).

The TEM analysis of the AgNP products was consistent with the absorption spectra. Figure 5 shows representative TEM images of AgNPs prepared with (a) 0.25, (b) 1, and (c) 10 mM NH_3_. At the optimal 1 mM concentration, the AgNPs formed had a mean size of 12.7 ± 4.9 nm. This size distribution was more monodisperse than a previous report of 10.3 ± 8.5 nm AgNPs synthesized by the femtosecond laser-irradiation of solutions containing AgNO_3_, NH_3_, and PVP as a capping agent [24], despite that we used no capping agent. Lower NH_3_ concentrations, for which the kinetics are faster, gave large amorphous, plate-like particles. Higher concentrations yielded agglomerations of particles, forming a variety of shapes, including star-like structures. Additional TEM images are presented in the supporting information, in Figures S3–S5.

## 4. Discussion

We have demonstrated in Section 3.1 that adding liquid ammonia to water reduces the amount of H_2_O_2_ produced in OB plasma (Figure 1c). This result indicates that ammonia acts as an effective OH· and H_2_O_2_ scavenger, which is in agreement with previous literature [3,4,5]. For ammonia solutions of ≥10 mM, an absorbance peak centered at 302 nm appeared during irradiation and slowly disappeared once the laser was turned off (Figure 2a,b). The 302 nm absorbance led us to believe that this species was peroxynitrite, ONOO^−^ [4,5,30,47,51,52,53,54]. In this section, we aim to understand the reactions involved between NH_3_ and OH· or H_2_O_2_ that occur during multiphoton absorption in water. The relevant reactions taken from the literature are summarized in Table 1.

Reactions involving ammonia in laser plasma are most likely initiated by hydroxyl radicals (Reaction 1 in Table 1), on the basis of previous reports that NH_3_ reacts with H_2_O_2_ only when a solution is irradiated with UV light to produce OH· [5]. Subsequent reactions of the NH_2_· radical can yield hydroxylamine and hydrazine (Reactions 2 and 3 in Table 1), which are known to reduce Ag^+^ ions and form AgNPs [55,56]. Although hydroxylamine and hydrazine were not detected in our experiments, they may be formed in small quantities and would be expected to contribute to Ag^+^ reduction. The NH_2_· radicals can also react with H_2_O_2_ and O_2_, eventually forming HNO_2_ and NO_3_^−^ (Reactions 4–8 in Table 1). NO_3_^−^ can scavenge hydrated electrons (Reaction 9), and both NO_2_^−^ and NO_3_^−^ form ONOOH or ONOO^−^ (Reactions 10–12). In the pH range in which we see what we believe is ONOO^−^ (pH 10.62–11.42; Supporting Information, Table S1), any ONOOH formed via Reaction 10 would quickly deprotonate to form ONOO^−^. Once formed, peroxynitrite is somewhat stable in pH values above its pK_a_ of 6.7 [52,57,58], and it decays spontaneously according to Reactions 13–19. To determine which reactions led to ONOO^−^ formation in the OB plasma, and to confirm that the species absorbing at 302 nm was ONOO^−^, additional experiments were conducted.

**Figure 6:**
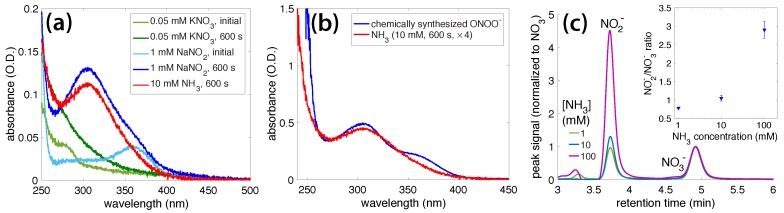
(**a**) Absorption spectra of initial and irradiated KNO_3_ and NaNO_2_ solutions, with irradiated NH_3_ for comparison; (**b**) Absorption spectra of irradiated NH_3_ and chemically synthesized ONOO^−^; (**c**) Ion chromatograms overlaid for 1, 10, and 100 mM NH_3_ solutions irradiated for 600 s. Peak heights normalized to NO_3_^−^ peak (labeled). Inset shows ${{\mathrm{NO}}_{2}}^{-}/{{\mathrm{NO}}_{3}}^{-}$ peak-height ratio as a function of NH_3_ concentration.

First, NaNO_2_ (1 mM) and KNO_3_ (0.05 mM) solutions, both with pH 10 (adjusted by adding KOH), were irradiated for 600 s under the same conditions as for our previous experiments. Figure 6a shows the initial and final absorbance spectra of the KNO_3_ solution (light and dark green) and NaNO_2_ solution (light and dark blue), along with the final absorption spectrum of the irradiated NH_3_ (red). While the irradiated KNO_3_ gave a broad increase in absorption below 350 nm, the irradiated NaNO_2_ produced an absorption peak nearly identical to that of the irradiated NH_3_ solution. This result indicated that NO_2_^−^, not NO_3_^−^, was the dominant ONOO^−^ precursor in our experiments. The presence of both OH· and H_2_O_2_ in the OB plasma was consistent with both Reactions 10 and 11 in Table 1, being possible pathways to ONOO^−^ formation.

Next, to verify that the species absorbing at 302 nm was peroxynitrite, we chemically synthesized ONOO^−^ by following the procedure in [47], involving a reaction between acidic H_2_O_2_ and alkaline nitrite. Figure 6b compares the absorption spectrum of our chemically synthesized ONOO^−^ to that of a 10 mM NH_3_ solution irradiated for 600 s. The spectra overlap at around 302 nm suggests that the unknown species was indeed peroxynitrite. The shoulder-peak in the chemically synthesized ONOO^−^ spectrum (Figure 6b), centered around 370 nm, was due to remaining NO_2_^−^ in the solution, and it matched the absorbance of the initial 1 mM NaNO_2_ solution in Figure 6a (light-blue spectrum).

Finally, to determine whether NO_2_^−^ and NO_3_^−^ were produced as the final products in our experiments, ion chromatography of the irradiated NH_3_ solutions was performed. Figure 6c shows overlaid ion chromatograms for the three irradiated NH_3_ solutions, normalized to the NO_3_^−^ peak height. The inset in Figure 6c shows the ${{\mathrm{NO}}_{2}}^{-}/{{\mathrm{NO}}_{3}}^{-}$ peak-height ratio as a function of the ammonia concentration, with a dramatic increase in the amount of NO_2_^−^ produced in the 100 mM NH_3_ solution. This result was consistent with previous reports of increased NO_2_^−^ production relative to NO_3_^−^ at high pH when NH_3_ and H_2_O_2_ solutions are irradiated with UV light [5,30]. This result may be explained by the solution pH (11.42; Table S1) approaching a pK_a_ of 11.6 for H_2_O_2_ [5,66]. Reactions 15–19 in Table 1 show the radical-mediated reactions that occur near the pK_a_ of H_2_O_2_, for which HNO_2_ is one of the products (Reaction 19).

Our observed ONOO^−^ decay rate, $k_{d}$ = 4.6 × 10^−4^ s^−1^, was significantly higher than the reported thermal decay rate of 1.3 × 10^−5^ s^−1^ and 3.2 × 10^−5^ s^−1^ at pH values of 13 and 12, respectively [63,65]. While these differences may have been caused by the lower pH range of 10.62–11.42 used in our experiments, it is also possible that the long-lived photolysis products in our experiments accelerated ONOO^−^ decomposition. The presence of H_2_O_2_ is known to speed up the degradation of peroxynitrite [47], which makes it the likely cause of the fast decay. We note that our ONOO^−^ decay experiments, reported in Figure 2b, were carried out in darkness, except for periodic UV-vis measurements of the absorption spectra every 300 s; thus photochemical decomposition according to Reaction 12 [52] was unlikely to have caused accelerated ONOO^−^ decay. In any case, the ultimate products of nitrite and nitrate (Figure 6c) suggested that our experiments followed similar mechanisms to those observed in previous reports on the conversion of ammonia to nitrite and nitrate through UV irradiation, in the presence of H_2_O_2_ [4,5,69]. It is difficult to interpret experimental observations related to the formation and decay mechanisms of peroxynitrite; thus findings are controversial [4,5,52,53,60,64,65,70]. A full discussion of the step-by-step mechanisms under various irradiation conditions is beyond the scope of this article and may be found elsewhere [5,52,60,70]. Our results demonstrate a new way to form this species and open avenues to its further investigation on ultrafast timescales.

**Figure 7:**
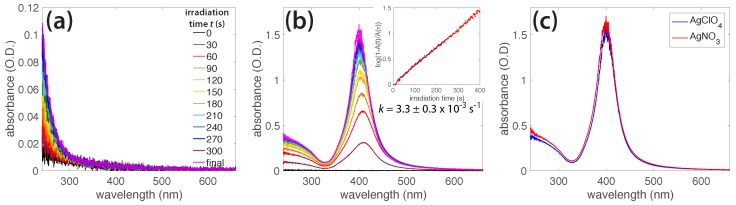
(**a**) Absorption spectra of AgClO_4_ in water irradiated for 600 s, showing no silver nanoparticle (AgNP) formation in the absence of ammonia; (**b**) Absorbance peak of AgNPs from AgClO_4_ (0.1 mM) in NH_3_ (1 mM) as it grew during irradiation; (**c**) Absorbance spectra of AgNPs formed from irradiating AgClO_4_ (red) and AgNO_3_ (blue) in 1 mM NH_3_.

The reactive species produced from ammonia photolysis, summarized in Table 1, assist the photochemical reduction of AgNO_3_ to generate AgNPs; the final AgNP morphology is determined in part by the ammonia concentration (Figure 5). No AgNP formation was observed when ammonia was not present (Supporting Information, Figure S1). Because nitrate is known to readily accept hydrated electrons (Reaction 9, Table 1) [30,38], the nitrate group of the AgNO_3_ precursor could hinder AgNP formation. To test this contention, we irradiated solutions containing AgClO_4_ (0.1 mM), a salt used in earlier radiolysis experiments because the ClO_4_^−^ ion does not scavenge hydrated electrons [39,40,41]. Figure 7a shows the absorption spectra of a AgClO_4_ solution irradiated for 600 s, with no AgNP formation. When ammonia (1 mM) was added to the AgClO_4_ solution and irradiated for 420 s, the 400 nm SPR absorption feature of the AgNPs grew with the irradiation time, at a rate constant of k = 3.3 ± 0.3 × 10^−3^ s^−1^ (Figure 7b), which was comparable to the rate constant of k = 4.1 ± 0.6 × 10^−3^ s^−1^ for AgNO_3_. Finally, the absorption spectra of the AgNPs prepared from the irradiation of both individual precursors, AgClO_4_ and AgNO_3_, displayed strong similarity (Figure 7c). These experiments supported the role of ammonia in driving the full reduction of Ag^+^ to form AgNPs and the negligible role that the nitrate group on AgNO_3_ plays in hindering AgNP formation in the absence of ammonia.

The similar AgNP formation rates and spectral properties observed for the 1 mM ammonia solutions of both Ag^+^ precursors suggest that the counterion has little effect on the Ag^+^ reduction rate. Instead, the reduction is controlled by reducing species produced from the photolysis of water and NH_3_ in OB plasma. On the basis of the high concentration of water (55 M) compared to NH_3_, the most likely reducing species is the hydrated electron, which can be formed at up to decimolar concentrations in OB plasma [17]. We are now in a position to explain the dependence of the observed Ag^+^ reduction kinetics on the NH_3_ concentration. Reduction kinetics are fast in low NH_3_ concentrations and slow as the NH_3_ molarity is increased (Figure 3).

At NH_3_ concentrations below 1 mM, the solution pH (5.71–8.42; Figure 3c and Table S1) is below the pK_a_ of NH_4_^+^ of 9.26, too low to support Ag^+^ complexation with ammonia to form Ag(NH_3_)_2_^+^ [42]. The fast reduction kinetics may therefore be attributed to the high reduction potential of free Ag^+^ as compared to the complex Ag(NH_3_)_2_^+^ [40,42]. These conditions result in the rapid formation of amorphous plate-like particles, as we observed at 0.25 mM (Figure 5a and Figure S3). At 1 mM NH_3_, the solution pH rose to 9.47, at which a small amount of NH_4_^+^ was present and most Ag^+^ was found as the Ag(NH_3_)_2_^+^ complex. Such complexation slows the reduction kinetics sufficiently to produce uniform spherical AgNPs (Figure 5b and Figure S4). As the NH_3_ concentration is further increased, a competing reaction mechanism can occur, in which the excess NH_3_ forms significant amounts of ONOO^−^. The observed decrease in the Ag^+^ reduction rate could be explained by the back-oxidation of Ag^0^, because ONOO^−^ acts as a strong oxidant [52,71]. Slow reduction led to the formation of large agglomerated AgNPs (Figure 5c and Figure S5), and no AgNPs were formed at all at NH_3_ concentrations above 20 mM. The absence of AgNP formation at high NH_3_ concentrations was consistent with increased ONOO^−^ production during irradiation and its accelerated decay after irradiation, in the presence of AgNO_3_ in 100 mM ammonia solution (Figure S2b,c). Collectively, these results indicate that for the optimal reduction of Ag^+^, finding a concentration of ammonia high enough to react with enough hydroxyl radicals and form the Ag(NH_3_)_2_ complex, but low enough to avoid excess peroxynitrite production, is key.

## 5. Conclusions

This work investigated the radical-mediated chemistry induced by a femtosecond laser plasma in aqueous solution. Adding liquid ammonia decreased the amount of H_2_O_2_ produced during water photolysis and formed a species that was determined to be peroxynitrite, ONOO^−^. The addition of NH_3_ to the aqueous solution was necessary to reduce Ag^+^ ions to form AgNPs, and 1 mM NH_3_ was determined to be the optimal concentration for making spherical AgNPs with mean diameters of 12.7 ± 4.9 nm. The extreme sensitivity of Ag^+^ reduction kinetics and the AgNP morphology demonstrated that understanding the reactions of the radical species produced in the laser plasma is crucial for NP synthesis using femtosecond laser irradiation methods. We anticipate that controlling the numbers of radical species in femtosecond laser plasma by varying the amount of NH_3_ added to aqueous solutions will help to better formation techniques for other metal NPs or alloyed metal NPs in the future.

## Figures and Tables

**Table 1:** Proposed reactions, rate constants, and references for photolysis of water and ammonia.

Reaction No.	Equation	Rate Constant (M^−1^ s^−1^)	Ref.
1	NH_3_ + OH· → NH_2_· + H_2_O	1 × 10^8^	[59]
2	NH_2_· + OH· → NH_2_OH	9.5 × 10^9^	[59]
3	2NH_2_· → N_2_H_4_		[60]
4	NH_2_· + H_2_O_2_ → ·NHOH + H_2_O	9 × 10^7^	[59]
5	NH_2_· + O_2_ → NH_2_O_2_·		[61]
6	NH_2_O_2_· + OH· → HNO_2_ + H_2_O		[61]
7	NO_2_^−^ + OH· → NO_2_· + OH^−^		[62]
8	NO_2_· + OH· → NO_3_^−^ + H^+^	1 × 10^10^	[62]
9	NO_3_^−^ + e_aq_^−^ → NO_3_^2−^		[30]
10	NO_2_^−^ + OH· → ONOOH	4.5 × 10^9^	[63]
11	NO_2_^−^ + H_2_O_2_ → ONOO^−^ + H_2_O		[47]
12	NO_3_^−^ + hν → ONOO^−^		[64]
13	ONOO^−^ + hν → NO_3_^−^	0.9 s^−1^	[65]
13’	ONOO^−^ → NO_3_^−^	~10^−5^ s^−1^	[63]
14	ONOO^−^ → NO_2_^−^ + 12O_2_		[52]
15	H_2_O_2_ → HO_2_^−^ + H^+^	pK_a_ = 11.6	[66]
16	HO_2_^−^ + OH· → OH^−^ + O_2_H·	7.5 × 10^9^	[67]
17	HO_2_^−^ + NH_2_· → NH_2_O_2_· + H^+^		[4]
18	NH_2_O_2_· → NO· + H_2_O		[60]
19	NO· + OH· → HNO_2_	8.9 × 10^9^	[68]

## Supplementary Materials

The following are available online.
