# Supplementary material for: Radical Chemistry in a Femtosecond Laser Plasma: Photochemical Reduction of Ag+ in Liquid Ammonia Solution
Source: Molecules. 2018 Feb 27;23(3):532. doi: 10.3390/molecules23030532 (PMC6017740; doi:10.3390/molecules23030532)

Supplementary file

Supporting Information for: Radical Chemistry in a Femtosecond Laser Plasma: Photochemical Reduction of Ag^+ in Liquid Ammonia Solution

Table S1. solution pH and fractional concentration of H_2O_2 for different ammonia concentrations.

$[NH_3]$ (mM)	pH	fractional yield of H_2O_2
0		1.00 ± 0.00
0.0625	5.71	
0.125	6.74	
0.25	6.98	
0.375	7.18	
0.5	7.67	
0.75	8.42	
1	9.47	0.91 ± 0.16
2.5	9.85	
5	10.25	0.61 ± 0.04
10	10.62	0.71 ± 0.05
20	10.93	
50	11.19	0.42 ± 0.05
75	11.34	
100	11.42	0.34 ± 0.03

Figure S1. $AgNO_3$ solution with no NH_3, (a) in-situ spectra over time shows that no particles are formed in this solution, (b) shows the $(TiSO_4)/H_2O_2$ spectra of $AgNO_3$ and $AgClO_4$ compared to one of just water irradiated under the same conditions. The fractional amount of H_2O_2 formed in the 0.1 mM $AgNO_3$ solution was 0.86 ± 0.11 and in the $AgClO_4$ was 0.85 ± 0.03.

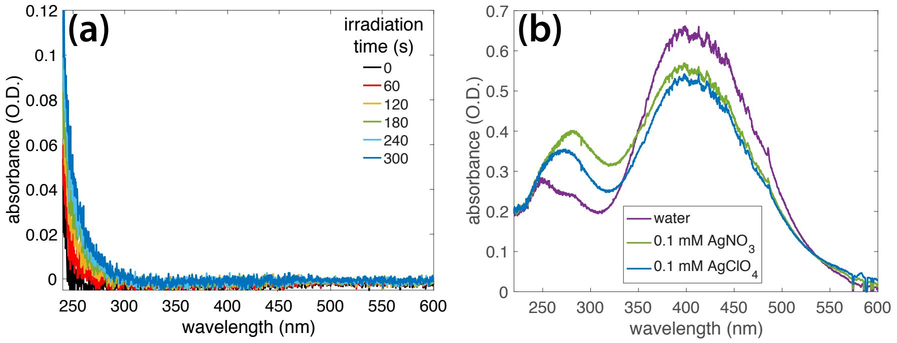

Table S2. All solutions contained 0.1 mM $AgNO_3$ and the indicated concentrations of NH_3. AgNP SPR growth rate constant k is shown for samples in which AgNPs formed

$[NH_3]$ (mM)	pH	k (s^{-1})
0	5.71	
0.0625	7.12	0.026 ± 0.002
0.125	7.19	0.029 ± 0.007
0.25	8.13	0.02 ± 0.01
0.375	8.83	0.010 ± 0.007
0.5	8.93	0.0049 ± 0.0008
0.75	9.18	0.0040 ± 0.0008
1	9.53	0.0041 ± 0.0006
2.5	10.23	0.0038 ± 0.0007
5	10.51	0.0037 ± 0.0008
10	10.78	0.0040 ± 0.0006
20	10.96	0.0030 ± 0.0005
50	11.22	
75	11.36	
100	11.44	

Table S3. Solutions contained 0.1 mM of silver salt, with the indicated concentrations of NH_3. The AgNP SPR growth rate constant k is shown for samples where AgNPs formed

$[NH_3]$ (mM)	Ag salt	pH	k (s^{-1})
0	$AgClO_4$	6.67 ± 0.44	
0	$AgNO_3$	6.15 ± 0.14	
1	$AgClO_4$	9.71 ± 0.10	0.0033 ± 0.0004
1	$AgNO_3$	9.53 ± 0.14	0.0041 ± 0.0006

Figure S2. Representative spectra of irradiated 100 mM NH_3 solutions with (a) and without (b) Ag. Average O.D. after 600 s with no Ag is 0.09 ± 0.01. with Ag: 0.11 ± 0.02. Comparative decay rates of the 302 nm peak over time (c).

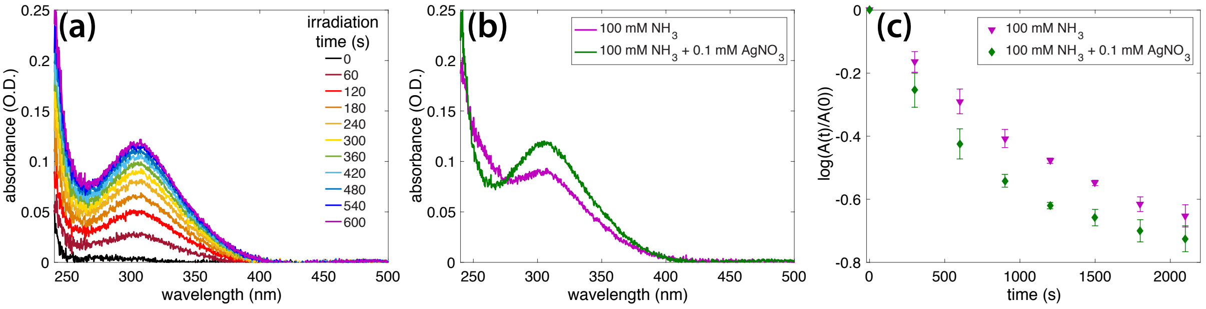

Figure S3. TEM image of AgNPs formed in 0.25 mM ammonia solution

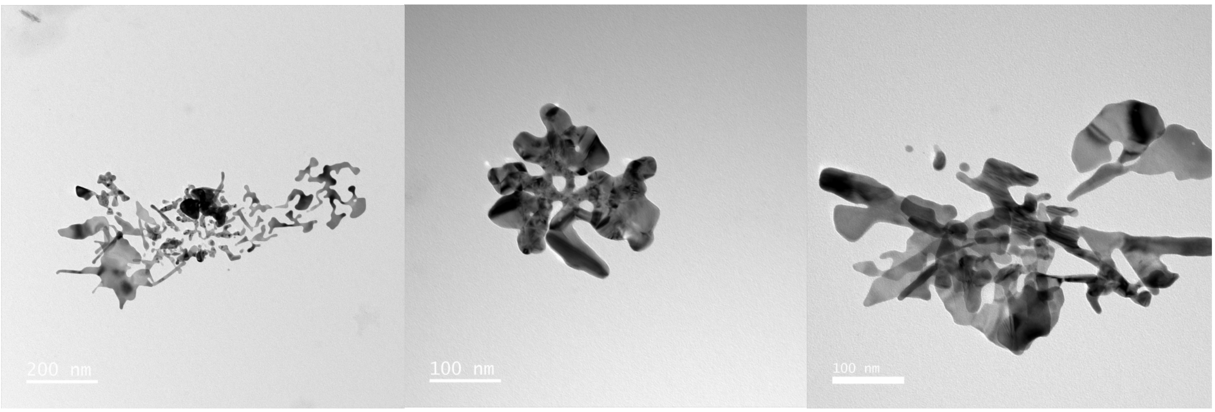

Figure S4. TEM image of AgNPs formed in 1 mM ammonia solution

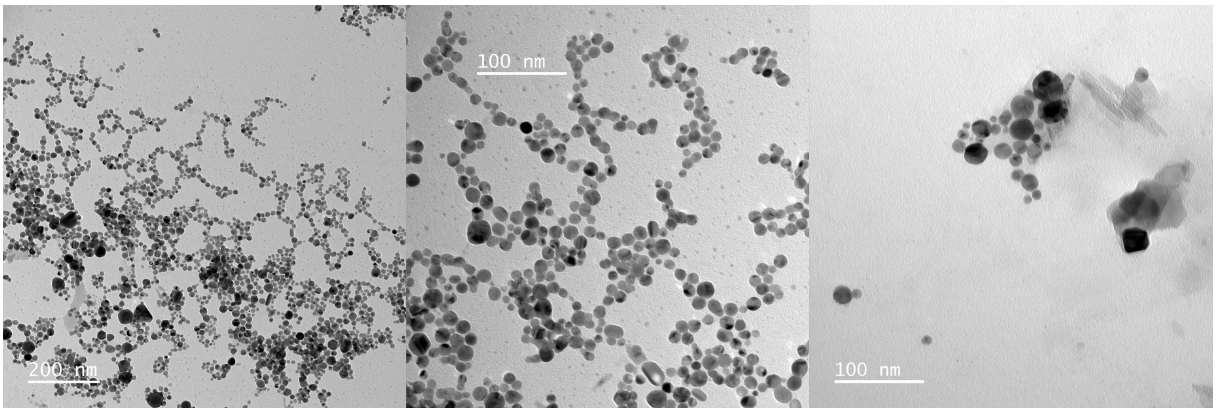

Figure S5. TEM image of AgNPs formed in 10 mM ammonia solution

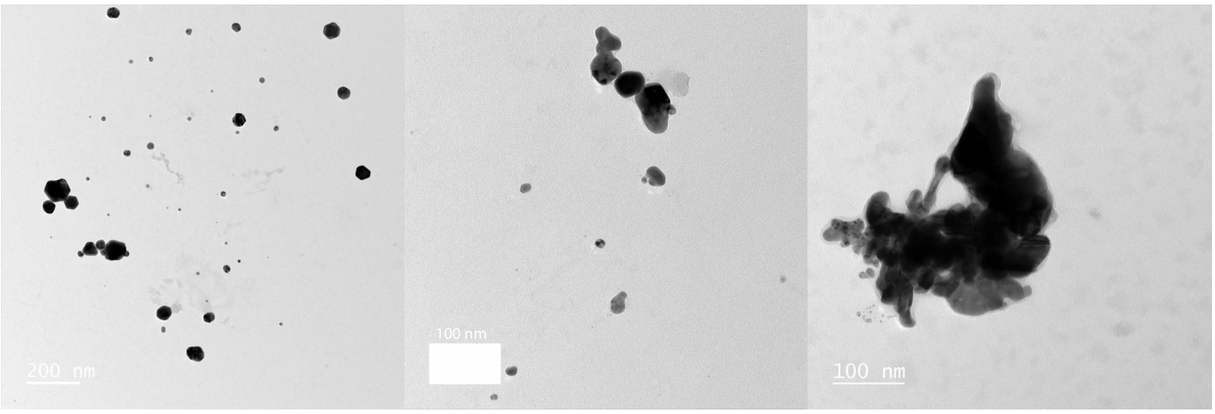

Supplement: Supplementary file 1 [file molecules-23-00532-s001.pdf]
